# The Twin-Arginine Translocation Pathway in α-Proteobacteria Is Functionally Preserved Irrespective of Genomic and Regulatory Divergence

**DOI:** 10.1371/journal.pone.0033605

**Published:** 2012-03-15

**Authors:** Pablo A. Nuñez, Marcelo Soria, Marisa D. Farber

**Affiliations:** 1 Instituto de Biotecnología, Instituto Nacional de Tecnología Agropecuaria (CICVyA-INTA), Buenos Aires, Argentina; 2 Cátedra de Microbiología Agrícola, Facultad de Agronomía, Universidad de Buenos Aires, INBA-CONICET, Buenos Aires, Argentina; University of Padova, Italy

## Abstract

The twin-arginine translocation (Tat) pathway exports fully folded proteins out of the cytoplasm of Gram-negative and Gram-positive bacteria. Although much progress has been made in unraveling the molecular mechanism and biochemical characterization of the Tat system, little is known concerning its functionality and biological role to confer adaptive skills, symbiosis or pathogenesis in the α-proteobacteria class. A comparative genomic analysis in the α-proteobacteria class confirmed the presence of *tatA*, *tatB*, and *tatC* genes in almost all genomes, but significant variations in gene synteny and rearrangements were found in the order Rickettsiales with respect to the typically described operon organization. Transcription of *tat* genes was confirmed for *Anaplasma marginale str. St. Maries* and *Brucella abortus* 2308, two α-proteobacteria with full and partial intracellular lifestyles, respectively. The tat genes of *A. marginale* are scattered throughout the genome, in contrast to the more generalized operon organization. Particularly, *tatA* showed an approximately 20-fold increase in mRNA levels relative to *tatB* and *tatC*. We showed Tat functionality in *B. abortus* 2308 for the first time, and confirmed conservation of functionality in *A. marginale*. We present the first experimental description of the Tat system in the *Anaplasmataceae* and *Brucellaceae* families. In particular, in *A. marginale* Tat functionality is conserved despite operon splitting as a consequence of genome rearrangements. Further studies will be required to understand how the proper stoichiometry of the Tat protein complex and its biological role are achieved. In addition, the predicted substrates might be the evidence of role of the Tat translocation system in the transition process from a free-living to a parasitic lifestyle in these α-proteobacteria.

## Introduction

Bacterial protein secretion systems are crucial for the interaction with both the environment and host cells, frequently targeting virulence determinants. Protein translocation across the bacterial cytoplasmic membranes and protein insertion in the membrane is achieved by one of two general pathways: the Sec protein-translocation system, which is the main route and exports unfolded proteins [1], and the twin-arginine translocation (Tat) pathway, which exports fully folded proteins [2]. Although much progress has been made in understanding the Tat system transport mechanism at a molecular level and in characterizing it biochemically, little has been learned about its biological role in bacteria, since it was discovered more than ten years ago.

The α-subdivision of Proteobacteria is a large and diverse group of Gram-negative microorganisms that show great variability in genome sizes and lifestyles and inhabit diverse ecological niches. Through multiple strategies, they establish both extra- and intracellular infection or associations with eukaryotes, yet many can also exist as free-living organisms [3]. Many plant and animal pathogens within this class use specialized secretion systems as molecular mechanisms to establish interactions with their host cells [3]. The ubiquity of these protein secretion systems correlates with highly variable composition and genome organizations that could compromise their functionality. Reduced genomes in Rickettsiales pathogens usually show the absence of orthologs genes or anomalous gene organization in gene clusters involved in the same biological pathway or protein complexes; in these cases, evidence of functional conservation is less conclusive or poorly known [4]–[7].

The Tat system is found in most bacteria, some archaea and thylakoid membranes of plant plastids [8]. Three functionally distinct components have been identified, namely TatA, TatB and TatC; however, their genomic organization is diverse. Gram-negative bacteria usually present these three components [9], [10] forming a heteromultimeric protein complex located in the inner membrane [2]. In contrast, TatB is absent in most Gram-positive bacteria and archaea, forming a minimal Tat Translocase (TatAC). The three genes are usually arranged in an operon (*tatABC*) in almost all the organisms with functional Tat systems described so far, while a few have their *tat* genes organized as individual transcriptional units [11]. The stoichiometry of the expression of the Tat subunits expression is critical for the activity of the Tat translocase [12]. The TatA protein is the most abundant component of the Tat system, present at an approximately 20-fold molar excess over the TatB and TatC components [2], thus, requiring higher expression levels than the other Tat proteins [13].

The TatA- and TatB-type proteins are sequence-related with a probable common ancestor [2]. Both comprise an N-terminal transmembrane α-helix followed by an adjacent amphipathic helix, connected by an interdomain hinge region and an unstructured C-terminal region of variable length [2], [14]. In organisms lacking *tatB*, it seems most likely that the TatA proteins retain both biological activities [15], [16]. Indeed, several bifunctional *Escherichia coli* TatA proteins that can bypass the requirement for TatB have been isolated in a study using an *in vivo* genetic screening for successful Tat transport [17]. TatC is the most conserved of the Tat proteins and sequence conservation is particularly strong within the six transmembrane (TM) domains [2], [18]. The signal peptides of the proteins exported by the Tat system share similar overall structures with the Sec-dependent signal peptides, but generally possess a twin-arginine (RR) motif in the n-region, a weak hydrophobic h-region, and a positively charged Sec avoidance signal just before the cleavage site. Recent studies have shown that a naturally occurring Lys-Arg (KR) motif, the R-N-R motif, or the variants KR, RK, and KK motifs in the n-region preserve the ability to mediate Tat translocation [19], [20]. The main function described for TatC is the primary recognition on the signal peptide and specifically for the RR motif [21].

Although the Tat system has been proved to be essential for virulence and symbiosis in several bacteria that interact with both plants and animals [22]–[29], few studies have addressed its role in the α-proteobacteria [30]–[32]. The aim of the present work was to explore the role of the Tat translocation system in α-proteobacteria by means of a genomic comparative analysis. In particular, we focused on *Brucella abortus* and *Anaplasma marginale*, well-known pathogenic α-proteobacteria, representative of facultatively and of obligately intracellular organisms, respectively.

## Materials and Methods

### Phylogenetic analysis and genome organization of *tatABC* genes

The amino acid sequences of 11 conserved proteins (RNA Pol ß and ß′, alanyl-tRNA synthetase, phenylalanyl-tRNA synthetase, arginyl-tRNA synthetase, EF-Tu, EF-G, RecA, GyrA, Gyrß and Hsp70) from 53 α-proteobacteria (Table S2) were downloaded from the RefSeq database of the NCBI and their identity confirmed using BLASTP (E-value = 10−5; Query coverage >70%). Protein sequences were aligned using ClustalW [33]. Poorly aligned positions of protein sequences were trimmed using Gblocks [34]. A phylogenetic tree was inferred from the concatenated alignments using the neighbor-joining method as implemented in the MEGA 4 software (JTT Model, 1000 bootstrap steps) [35]. For analyzing the presence of the Tat genes in the 53 α-proteobacteria genomes, the putative *tatA, tatB* and *tatC* genes were identified using BLASTP against well-documented *tat* genes. The genome organization of the Tat genes was visualized using NCBI's Mapviewer (http://www.ncbi.nlm.nih.gov/projects/mapview/) or KEGG (http://www.genome.jp/kegg/). The *A. marginale str. St. Maries tatA* (missing in the annotated genome, flanked by AM392 and AM394), *tatB* (AM476) and *tatC* (AM740) genes and the *Brucella melitensis biovar abortus 2308 tatA (BAB1_0901), tatB* (*BAB1_0902*) and *tatC* (*BAB1_0903*) genes, were displayed with the Circular Genome Viewer (CGView) [36]. The GenBank accession number for the nucleotide sequence of *tatA* from *A. marginale str. Salta* is JQ409478 (100% sequence identity with *tatA* from *A. marginale str. St. Maries)*. The *A. marginale str. St. Maries* gene AM476 (*tatB*) was annotated as hypothetical protein.

### RNA isolation and reverse transcription RT-PCR

Total RNA from *A. marginale str. Salta* [37] was obtained from 2-ml frozen whole blood stabilate of an infected bovine whereas total RNA from *B. melitensis biovar abortus 2308* was obtained from a 3-day culture in TBS medium (BD, USA) at 37°C and 200 rpm. The RNAeasy kit (Qiagen, CA, USA) was used according to the manufacturer's instructions for mRNA extraction. The concentration and purity of the RNA were determined by measuring the A260/A280 ratio with a Nanodrop ND-1000 (NanoDrop Technologies Inc, USA). Then, 1 µg of the extracted RNA was treated with 1 U of DNase I amplification grade (Invitrogen, USA) at room temperature for 30 min. DNase I was then inactivated by addition of 1 µl of 25 mM EDTA and subsequent heating at 65°C for 10 min, and 1 µl (3 µg/µl) Random primers (Invitrogen, USA), 1 µl DNTPs 10 mM (Promega, USA) and MilliQ water (Millipore) up to 13 µl was added to the DNase I-treated RNA. The mixture was heated for 5 min at 65°C and then chilled for 5 min in ice-water. After addition of 1 µl SuperScript III Reverse Transcriptase together with 4 µl First strand Buffer (Invitrogen, USA) and 1 µl 0.1 M DTT, the reaction mixture was incubated for 5 min at room temperature, followed by 60 min at 50°C. The reaction was terminated by heating at 70°C for 15 min. To monitor DNA contamination, an identical reaction mixture was prepared without RT Super Script III.

### Plasmid constructions

Genomic DNA from *A. marginale str. St. Maries* was kindly provided by Dr. Guy Palmer (Department of Veterinary Microbiology and Pathology, Washington State University, Pullman, Washington). DNA from *B. abortus 2308* was prepared from pure cultures by three cycles of freeze-thawing [38] from heat-inactivated biomass (bacteria were heated at 99°C for 10 min and centrifuged for 2 min at 13,000 g). Then, 5 µl of the supernatant were used for PCR assays. Specific primers were designed (Table S1), and standard protocols were used for PCR using DNA extracted from both organisms to amplify the *tatA*, *tatB* and *tatC* genomic sequences. The PCR fragments were cloned into pTOPO2.1 (Invitrogen, USA) prior to subcloning in the pUNIPROM plasmid [39] under the control of the *E. coli tat* promoter [40] and sequenced to confirm integrity. A list of pUNIPROM plasmids used in this study is shown in Table 1. During all cloning steps, *E. coli* strains were grown aerobically in LB medium using standard concentrations of antibiotics.

**Table 1:** Strains and plasmids used in this study.

Bacterial Strains	Genotype	Source
MC-4100-P	Km^r^	T. Palmer Lab
JARVIG-P	Δ*tatA*Δ*tatE*; Km^r^	T. Palmer Lab
BOD-P	Δ*tatB*; Km^r^	T. Palmer Lab
BILKO-P	Δ*tatC*; Km^r^	T. Palmer Lab
DADE-P	Δ*tatABC*; Km^r^	T. Palmer Lab
DH5α		Promega
**Plasmids**		
pUNIPROM	Amp^R^	T. Palmer Lab
pUNIPROM_AmTatA	Amp^R^	This work
pUNIPROM_AmTatB *(AM476)*	Amp^R^	This work
pUNIPROM_AmTatC (*AM740*)	Amp^R^	This work
pUNIPROM_BaTatA (*BAB1_0901*)	Amp^R^	This work
pUNIPROM_BaTatB (*BAB1_0902*)	Amp^R^	This work
pUNIPROM_BaTatC (*BAB1_0903*)	Amp^R^	This work
pFAT415	Amp^R^	T. Palmer Lab
pFAT416	Amp^R^	T. Palmer Lab
pFAT417	Amp^R^	T. Palmer Lab

### Bacterial strains and growth conditions

Plasmid constructions were used to transform competent *E. coli tat* mutants (MC4100-P, JARVI6-P, BOD-P, BILK0-P and DADE-P; Table 1) [10], [40] for complementation assays. The *E. coli* mutant strains JARVI6-P (Δ*tatA*), BOD-P (Δ*tatB*) and BILK0-P (Δ*tatC*) [40] were complemented with pUNIPROM vectors containing *tatA, tatB* and *tatC* from *A. marginale str. St. Maries* and *B. abortus 2308*, respectively, using standard transformation protocols. The mutant strains complemented with the pUNIPROM empty vector and the DADE-P strain (Δ*tatABC*) were used as negative controls. Wild type MC4100-P and *E. coli tat* mutants complemented with pUNIPROM containing native *E. coli tatA, tatB* and *tatC*, pFAT415, 416 and 417 [10], respectively, were used as positive controls. To assess functionality of the heterologous *tat* genes, the control and complemented strains were grown under different selective conditions: (i) 2% SDS: cells were grown in liquid LB medium overnight at 37°C and then tested in LB agar plates supplemented with 2% SDS [41], or in liquid medium plus 2% SDS, measuring growth by optical density at 600 nm for several hours [40]; (ii) anaerobic conditions: cells were grown overnight at 37°C and tested in M9 minimal medium agar plates supplemented with 0.5% glycerol and 0.4% trimethylamine-N-oxide (TMAO) and incubated in a gas jar under a hydrogen/carbon dioxide atmosphere [40]; (iii) TMAO reductase assay: subcellular fractions for TMAO reductase activity measurements were prepared from small (30 ml) cultures incubated overnight without shaking at 37°C in liquid LB TMAO/glycerol medium supplemented with 50% glycerol and 20% TMAO under anaerobic conditions. Periplasmic fractions were obtained by using the cold osmotic shock method [40], [42]. Protein concentration in the periplasmic fraction was measured after the enzymatic assay (Pierce, Thermo Scientific, USA). TMAO benzyl viologen oxidoreductase activity was measured as described previously (Thermo MultiSkan Spectrum, Thermo Scientific, USA) [43].

### Microscopy

Overnight cultures of complemented *E. coli* mutant and control strains were diluted 1∶100 in LB and grown at 37°C until a 600 nm optical density of 0.6–0.8 was reached [44]. The cells were examined with phase-contrast microscopy using a Leica TCS-SP5 (Leica Microsystems GmbH, Wetzlar, Germany) spectral laser confocal microscope using a 63× objective (HCX PL APO CS 63.0×1.20 WATER UV).

### RT-PCR and Quantitative real-time PCR

Primers were designed using Primer Express Version 2.0 (Applied Biosystems) (Table S1). The internal control genes tested were *groEL* for *A. marginale* and *rpll* for *B. abortus 2308*. Ten-fold serial dilutions of the cDNA were used in the real-time PCR to construct the standard curve and calculate the efficiency for each set of primers. Assays with a correlation coefficient (r*)* value of >0.99 were considered acceptable. Quantitative PCR was performed with a real-time instrument (ABI PRISM®, 7000, Sequence Detection System, Applied Biosystems) using a Quantitect SYBR green (QIAGEN). Results were analyzed using the relative expression software tool (REST) for group-wise comparisons for the *tatA, tatB* and *tatC* genes and statistical analysis of the relative *tatA, tatB* and *tatC* expression rates [45].

### *In silico* prediction of Tat substrates

Potential Tat substrates in the protein set coded by the annotated genomes of *Anaplasmataceae* (12 species) and *Brucellaceae* (10 species) families, available at NCBI, were searched using the three existing programs for Tat signal prediction: TatP (http://www.cbs.dtu.dk/services/TatP/; [46], TATFIND (http://signalfind.org/tatfind.html; [47] and PRED-TAT (http://www.compgen.org/tools/PRED-TAT/ [48]. The TatP program combines the search of patterns of amino acid sequences with two neural networks one trained to detect cleavage sites and the other to determine whether an amino acid belongs to the Tat signal peptide or not. TATFIND predicts Tat sites by searching patterns of amino acid sequences and hydrophobicity. PRED-TAT applies Hidden Markov models to predict and discriminate between Sec and Tat signal peptides. TatP and TATFIND were run from their respective servers. The authors of PRED-TAT provide a repository of pre-processed bacterial genomes from which we extracted the predicted Tat targets for the *Anaplasmataceae* and *Brucellaceae* proteins (http://www.compgen.org/tools/PRED-TAT/supplement/genomes). We considered that a protein contained a putative Tat signal if it was predicted by at least two of the software programs.

## Results

### Organization and distribution of Tat genes among the α-proteobacteria

**Figure 1:**
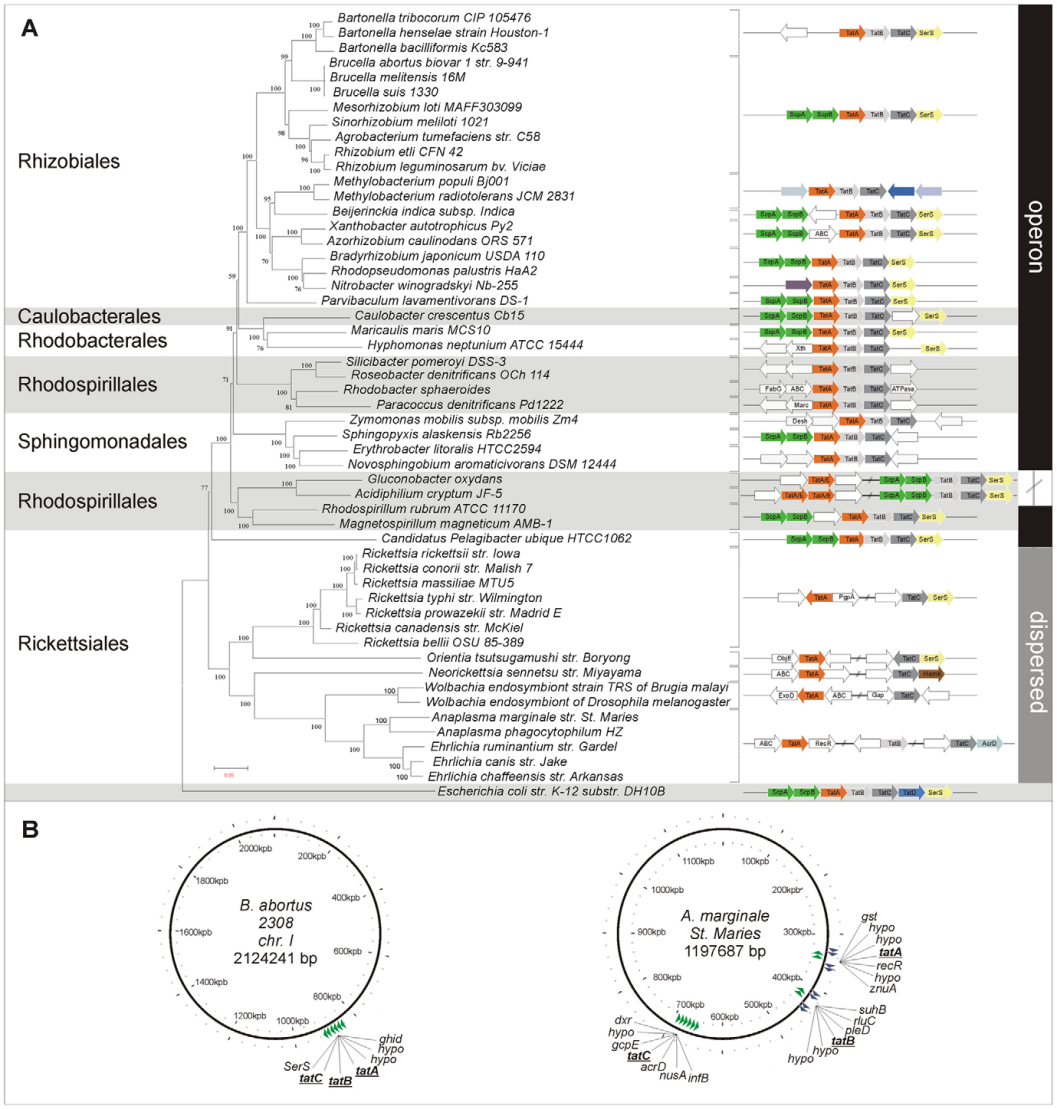
Phylogenetic analysis and genome organization of *tatABC* genes. (A) Left side panel: Phylogenetic tree construction using neighbor-joining method (NJ-JTT, 1000 bootstrap) with MEGA.4 of 53 bacterial species based on the concatenated alignment of 11 conserved proteins (RNA Pol ß and ß′, alanyl-tRNA synthetase, phenylalanyl-tRNA synthetase, arginyl-tRNA synthetase, EF-Tu, EF-G, RecA, GyrA, Gyrß, Hsp70). Right side panel: Genome organization of *tatABC* genes. (B) *tatA, tatB* and *tatC* genes in *Anaplasma marginale str. St. Maries*, and *Brucella melitensis biovar abortus 2308* (Chr I) were displayed and visualized with the Circular Genome Viewer (CGView). Orthologs genes for *tatA*, *tatB* and *tatC* were found in almost all the genomes studied, with the exception of species analyzed from the genera *Rickettsia, Neorickettsia, Orientia* and *Wolbachia* that lacked the *tatB* gene. Three different main organizations according to operon structure preservation were found; one with the commonly described operon organization, another one with a partially dispersed organization (*tatA* locus maps in a different location from that of the *tatBC* operon in the same strand), and a completely scattered distribution for *tat* genes in well-separated location of the circular genomes in several genera of the order Rickettsiales. Organisms lacking a *tatB* homolog, with the exception of *Neorickettsia* species, encoded *tatA* and *tatC* in different genome strands. The bracket indicates the organisms that share a common *tat* genes organization.

To explore the genomic architecture of the Tat system in the α-proteobacteria, we carried out a comparative analysis by selecting 53 genomes which represent all orders within the class. We confirmed the presence of the *tatA*, *tatB* and *tatC* orthologs genes in 42 out of the 53 genomes studied, while in the remaining 11 genomes we only detected the *tatA* and *tatC* orthologs (Figure 1A). Previous results from our laboratory using a phoA fusion system for experimentally detecting signal peptides in *A. marginale* allowed us to identify an open reading frame (ORF) of 171 nucleotides flanked by the loci tags AM392 and AM394, omitted in the annotation of the *A. marginale str. St. Maries* genome [49]. Translation of the ORF rendered a predicted protein of 53 amino acids with a highly conserved N-terminal region identified as the *tatA* gene (Figure S1). Likewise, the *tatA* gene is missing in *Ehrlichia ruminantium str. Gardel* annotated genome [50]. Indeed, ORFs of small size like *tatA* are prone to misidentification via standard genome automation methods. In addition, using tblastn we confirmed that species from the genera *Rickettsia, Neorickettsia, Orientia* and *Wolbachia* lacked the *tatB* gene. The genomes were sorted in three different groups according to operon structure conservation and synteny (Figure 1A). The first group encompasses all the genomes analyzed from Rhizobiales (including *B. abortus*), Caulobacterales, Rhodobacterales, Sphingomonadales and two species from the order Rhodospirillaes, *Rhodospirillum rubrum ATCC 11170* and *Magnetospirillum magenticum AMB-1*. They all have the commonly described organization with the three genes as part of a single operon. The second group has a partially dispersed organization, in which the *tatA* locus maps in a different location from that of the *tatBC* operon but codes in the same strand. This group consists of two members: *Gluconobacter oxydans* and *Acidiphilium cryptum JF-5*, both from the Acetobacteraceae family. The last group included several genera of the order Rickettsiales: *Anaplasma*, *Ehrlichia*, *Neorickettsia* (family Anaplasmataceae), *Wolbachia* and *Rickettsia* (family Rickettsiaceae), and showed a completely scattered distribution of the *tat* genes in well-separated locations of the circular genomes (Figure 1B). Organisms lacking a *tatB* homolog, with the exception of *Neorickettsia* species, also encode *tatA* and *tatC* in different genome strands.

### Tat genes transcription analysis

**Figure 2:**
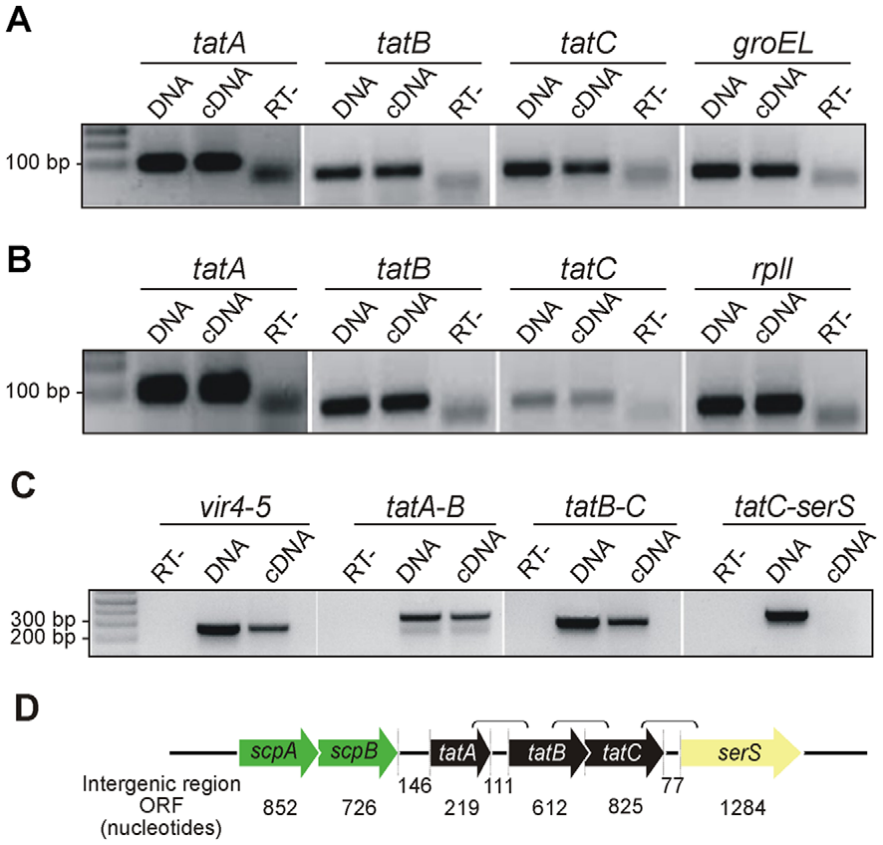
Transcription of *tatA, tatB* and *tatC* genes and polycistronic mRNA of *tatABC* genes in *Brucella abortus*. (A) RT-PCR of *A. marginale*; the *groEL* gene was used as housekeeping gene. (B) RT-PCR of *B. abortus*; the *rpll* gene was used as housekeeping gene. (Bands below 100 bp in the RT (−) lanes correspond to primer dimmers). (C) Operon transcription organization for *B. abortus*. Operon Vir4-5 was used as control. (D) Genomic structure of *B. abortus tatABC* genes and intergenic regions. Specific primers for RT-PCR designed to amplify *tatAB*, *tatBC*, *C-SerS* and *Vir4-5* are shown in parentheses. Results confirmed transcription of the three genes in both organisms. Using RT-PCR with primers that amplified from the 3′ region of one ORF to the 5′ region of a contiguous ORF, we confirmed a polycistronic mRNA transcript for *B. abortus tatABC* genes, excluding the contiguous *serS*.

To confirm the expression of the complete translocation system components, the transcription of the *A. marginale tatA* (JQ409478), *tatB* (AM476) and *tatC* (AM740) genes and *B. abortus tatA* (*BAB1_0901), tatB (BAB1_0902) and tatC (BAB1_0903)* was assessed by reverse transcription PCR assays. Results were positive for the three genes in both organisms (Figure 2A, 2B). We performed RT-PCR from *B. abortus* cDNA using a forward oligonucleotide matching the 3′ region of the upstream ORF and a reverse oligonucleotide specific to the 5′ region of a contiguous ORF. We detected an amplicon of the expected size when using the specific primers for the contiguous genes *tatA–tatB* and *tatB–tatC*. Conversely, no amplicon was detected when using specific oligonucleotide for *tatC*-*serS*. In this way, we confirmed the polycistronic mRNA for the *tat* genes in *B. abortus* (Figure 2C, 2D).

### Heterologous expression of Tat A, B and C proteins

Since we corroborated the transcription of the three components of the Tat system in both bacterial species, we tested protein functionality through a complementation test in *E. coli*. Individual Tat subunits were tested for their ability to substitute for the absence of the cognate *E. coli* Tat component and thus form functional Tat translocases with *E. coli* Tat proteins. The *E. coli* mutants JARV16-P (Δ*tatA*; Δ*tatE*), BOD-P (Δ*tatB)*, and BILK0-P (Δ*tatC*) were every time individually complemented with the *tatA, tatB* or *tatC* genes from both *A. marginale* and *B. abortus*. We used four different tests to assess the functionality of the Tat system (see below) [40], [44], [51].

### Chain-forming phenotype

**Figure 3:**
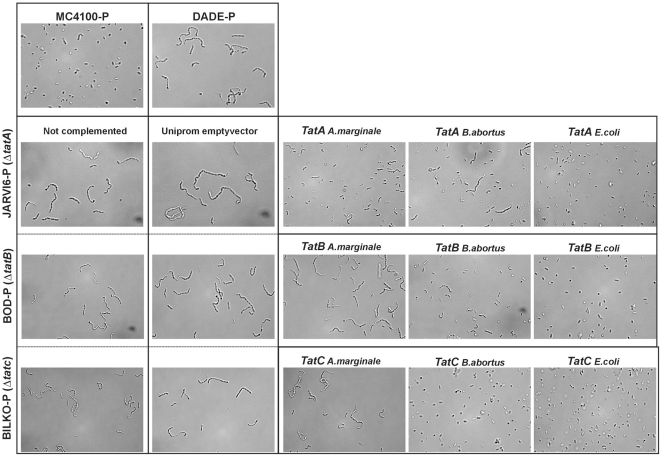
Functionality of TatA, B or C by analyzing their ability to complete cellular division. Phase-contrast microscopy of control cells M4100-P, DADE-P, *E. coli tat* mutants (JARVI6-P, BOD-P and BILK0-P), complemented with the pUNIPROM empty vector and complemented with experimental heterologous genes from *A. marginale, B. abortus* or *E. coli* native genes. Cells were examined by Leica TCS-SP5 Laser Confocal Microscope and each picture was taken at 63X magnification. Cells complemented with *A. marginale* genes revealed complementation only for the TatA subunit. In contrast, in the case of *B. abortus*, TatA failed to restore the complete Tat activity and TatB and TatC restituted Tat functionality.

Since the *amiB* gene encodes a Tat-dependent secreted cell wall amidase involved in cleaving the murein septum during cell division [52], the Tat mutants resulted in a high frequency of cell chains between 6 and 24 cells in length after a growth cycle [44], [51]. The *E. coli* mutants (JARVI6-P, BOP-P and BILK0-P) without plasmids or complemented with the p-UNIPROM empty vector showed a chain-forming phenotype (Figure 3). On the other hand, cells complemented with *E. coli* native genes completely restored the Tat system functionality, leading to a single-cell phenotype due to correct cleavage of the septum. Cells complemented with *A. marginale tatA, tatB* or *tatC* genes rendered a single-cell phenotype only for the *tatA* gene. In contrast, for *B. abortus* we observed the opposite results: a single-cell phenotype when complemented with *B. abortus tatB* and *tatC*, but preservation of the anomalous phenotype when complemented with *B. abortus tatA* (Figure 3; Table 2).

**Table 2:** Results of Tat subunits functionality.

*Anaplasma marginale str. St. Maries*				
Test	Cell-chain phenotype	SDS-resistant phenotype	Anaerobic-TMAO growth	TMAO reductase activity (%)^a^
TatA	yes	yes	yes	25,48*
TatB	no	no	yes	32,33
TatC	no	no	no	13,03
***Brucella melitensis biovar abortus 2308***				
TatA	no	no	yes	41,37*
TatB	yes	yes	yes	41,67
TatC	yes	yes	yes	78,82*

a Activity relative to positive control.

* p<0.05 statistically significant.

### SDS-resistance phenotype, anaerobic-TMAO growth and TorA activity

*E. coli* depleted of any of the Tat components experienced a pleiotropic cell envelope defect due to an inability to export two Tat-dependent periplasmic amidases (AmiA and AmiC) that are involved in cell wall integrity. As a consequence, mutant strains are unable to grow on solid media in the presence of 2% SDS [40], [41], [44]. On the other hand, wild type *E. coli* is able to grow anaerobically using trimethylamine-*N*-oxide (TMAO) as an electron acceptor due to two enzymes that are known to be translocated to the periplasm by the Tat system: the soluble periplasmic TMAO reductase (TorA) and the membrane-bound protein dimethylsulphoxide reductase (DmsABC) [53].

**Figure 4:**
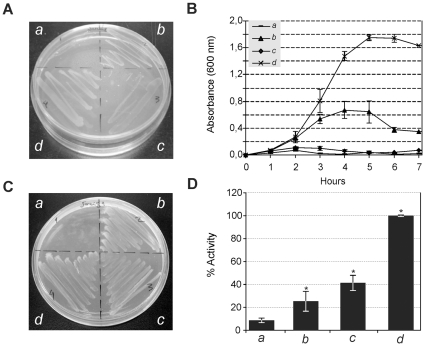
Functionality of TatA subunits. Complementation of the *E. coli* Δ*tatA–*Δ*tatE* (JARVI6-P) with heterologous *tatA* genes from *A. marginale (b), B. abortus (c)* and *E. coli (d*). The UNIPROM empty vector (*a*) was used as a negative control. Strains were grown on: (A) LB medium agar plates containing 2% SDS. (B) LB liquid medium containing 2% SDS. (C) Agar plates under anaerobic conditions with minimal medium supplemented with glycerol as a carbon source and TMAO as sole electron acceptor. (D) TMAO reductase activity from periplasmic fractions. *100% activity is taken as that determined from the periplasmic fraction of JARVI6-P carrying the *E. coli tatA* gene (pFAT415*)*. Error bars represent the standard error of the mean of three independent experiments. (*) p<0.01 ANOVA, LSD-Fisher, Statistic 6.0. Complementation of JARVI6-P with *A. marginale tatA* gene resulted in significant restoration of Tat system function. In contrast, *B. abortus* TatA failed to restore functionality under these growth conditions. TatA of both organisms restored Tat functionality, showing growth in the M9, TMAO agar plates and also a statistically significant TMAO reductase activity of 25% and 41% for *A. marginale* and *B. abortus*, respectively.

**TatA:** As shown in Figure 4, expression of *A. marginale* TatA proteins in the *E. coli* JARV16-P (Δ*tatA*; Δ*tatE*) mutant strain resulted in significant restoration of the Tat system function under the presence of SDS (Figure 4A, 4B), suggesting that it is capable of heterologous interaction with the *E. coli* TatBC proteins to form a functional protein complex. In contrast, *B. abortus* TatA failed to restore functionality under this growth condition (Figure 4A, 4B). The TatA protein of both organisms showed robust growth with TMAO as sole terminal electron acceptor (Figure 4C) and had a significant TMAO reductase (TorA) activity in the periplasmic fraction of 25% and 41% for *A. marginale* and *B. abortus*, respectively (Figure 4D).

**Figure 5:**
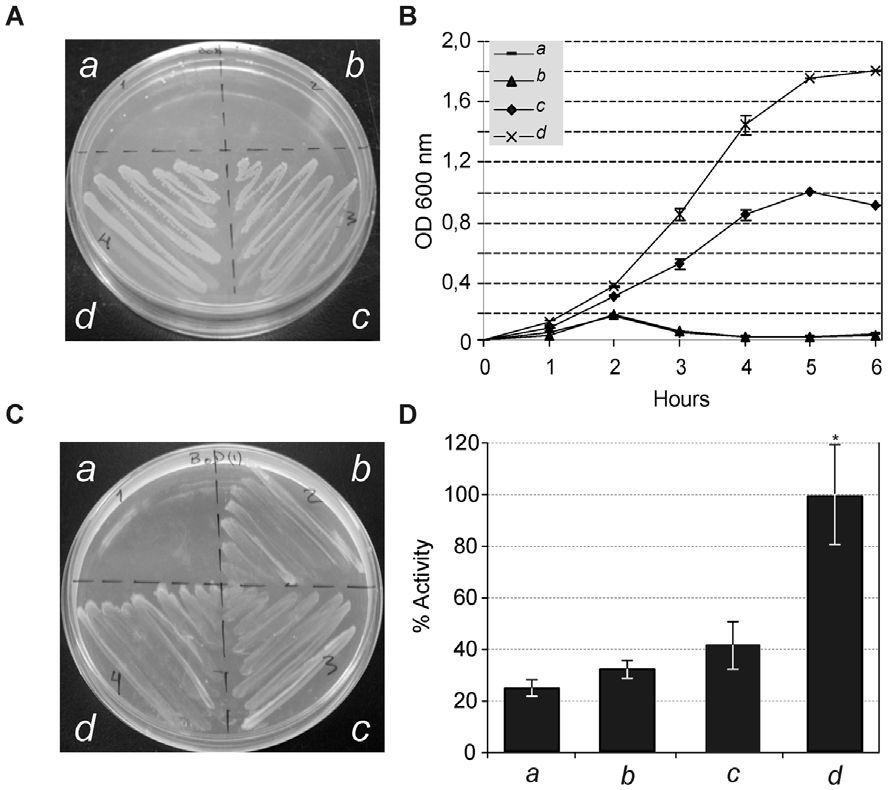
Functionality of TatB subunits. Complementation of the *E. coli* Δ*tatB* (BOD-P) with heterologous *tatB* from *A. marginale (b), B. abortus (c)* and *E. coli (d*). The UNIPROM empty vector (*a*) was used as a negative control. Strains were grown on: (A) LB-medium agar plates containing 2% SDS. (B) LB liquid medium containing 2% SDS. (C) Agar plates under anaerobic conditions on minimal media with glycerol as a carbon source and TMAO as sole electron acceptor. (D) TMAO reductase activity from periplasmic fractions. *100% activity is taken as that determined from the periplasmic fraction of BOD-P carrying the *E. coli tatB* gene (pFAT416*)*. Error bars represent the standard error of the mean of three independent experiments. (*) p<0.01 ANOVA, LSD-Fisher, Statistic 6.0. TatB subunit of *A. marginale* failed to restore ability to grow in the presence of 2% SDS in LB medium, since non-significant growth can be observed either in agar or liquid medium conditions. However, it was sufficient to restore viability under anaerobic conditions, showing levels of TMAO reductase activity higher than those of the negative control, although it was not statistically significant. For *B. abortus*, the TatB subunit completely restored resistance under 2% SDS and anaerobic conditions; however, similarly to TatB of *A. marginale*, TorA activity was higher, but not statistically significant, than the negative control.

**TatB:** The TatB subunit of *A. marginale* failed to restore the ability to grow in the presence of 2% SDS in LB medium, since no significant growth was observed either in agar or liquid medium conditions (Figure 5A, 5B). However, it was sufficient to restore viability under anaerobic conditions (Figure 5C), showing levels of TMAO reductase activity higher than the negative control (empty vector), although not statistically significant (Figure 5D). In the case of *B. abortus*, the TatB subunit completely restored resistance under 2% SDS (Figure 5A, B) and anaerobic conditions (Figure 5C); however, similarly to TatB of *A. marginale*, TorA activity was higher, but not statistically significant referred to the negative control (Figure 5D).

**Figure 6:**
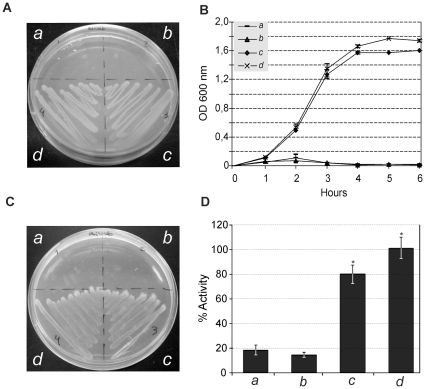
Functionality of TatC subunits. Complementation of the *E. coli* Δ*tatC* (BILK0-P) with heterologous *tatC* from *A. marginale* (*b*), *B. abortus* (*c*) and *E. coli* (*d*). The UNIPROM empty vector (*a*) was used as a negative control. Strains were grown on: (A) LB-medium agar plates containing 2% SDS. (B) LB liquid medium containing 2% SDS. (C) Agar plates under anaerobic conditions on minimal media with glycerol as a carbon source and TMAO as sole electron acceptor. (D) TMAO reductase activity from periplasmic fractions. *100% activity is taken as that determined from the periplasmic fraction of BILK0P carrying the *E. coli tatC* gene (pFAT417*)*. Error bars represent the standard error of the mean of three independent experiments. (*)p<0.01 ANOVA, LSD-Fisher, Statistic 6.0. TatC of *A. marginale* was unable to restore Tat functionality either under 2% SDS or anaerobic conditions, and no detectable levels of TMAO reductase were measured in the periplasmic fractions. In contrast, TatC of *B. abortus* completely restored capacity to grow under both selective conditions, and higher levels of TMAO reductase were recorded in the periplasmic fractions.

**TatC:** TatC of *A. marginale* was unable to restore Tat functionality either in the presence of 2% SDS or under anaerobic conditions, and no detectable levels of TMAO reductase were measured in the periplasmic fractions. In contrast, TatC of *B. abortus* completely restored the capacity to grow under both selective conditions, and higher levels of TMAO reductase were recorded in the periplasmic fractions (Figure 6A, 6B, 6C, 6D).

### *tatA, tatB* and *tatC* mRNA transcript levels

**Figure 7:**
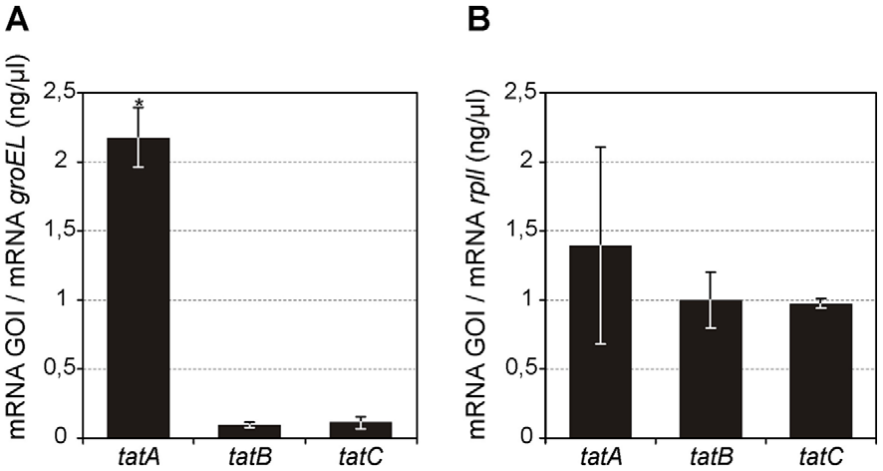
*TatA, tatB* and *tatC* mRNA transcript levels. Real time q-PCR for determining the mRNA abundance of *tatA, tatB* and *tatC* genes for both organisms, (GOI: gene-of-interest) (A) *A. marginale str. St. Maries* (B) *Brucella melitensis biovar abortus 2308*. (*) p<0.001, Pair Wise Fixed Reallocation Randomization Test. *A. marginale tatA* showed a 23- and 19-fold increase in expression relative to *tatB* and *tatC*, respectively. For *B. abortus* the expression rate did not differ between *tatA, tatB* and *tatC genes*. *A. marginale tatA* showed a 23- and 19-fold increase in expression relative to *tatB* and *tatC*, respectively. In the case of *B. abortus*, the mRNA levels did not differ between the *tatA, tatB* and *tatC* genes.

Taking into consideration that *A. marginale* TatA and TatB components were able to restore the *E. coli* Tat system functionality, we decided to analyze the preservation of the expected stoichiometry of the TatABC components, which has been described as critical for export function [2]. To study the transcript levels of the *tat* genes, we performed real time PCR to quantify the mRNA abundance of the three genes in both organisms. *A. marginale tatA* showed a 23- and 19-fold increase in expression relative to *tatB* and *tatC*, respectively, equivalent to the expected stoichiometry of functional protein translocase machinery. On the other hand, for *B. abortus*, the mRNA abundance did not differ between the *tatA, tatB and tatC* genes, as expected for polycistronic mRNA (Figure 7).

### Tat substrates prediction

After demonstrating Tat system functionality in the microorganism selected, we searched *in silico* for potential translocation system substrates. The predicted protein sets from both *Brucellaceae* and *Anaplasmataceae* species were scanned using the three existing algorithms designed to detect the N-terminal Tat-signal peptide. We identified putative Tat-dependent secreted proteins in the families *Brucellaceae* (10 species) and *Anaplasmataceae* (12 species). We considered as potential Tat substrates those which gave a positive result for a possible Tat signal sequence with at least two of the three software programs. The putative Tat substrates were grouped based on functional categories from the Cluster of Orthologs Groups (COGs). Nevertheless, the predicted Tat-secreted proteins ought to be experimentally validated to become true substrates.

Three positive substrates with COG definitions associated were identified for *Anaplasmataceae* (Table 3). We identified the *Rieske Fe-S* protein (COG0723) represented in 8 out of the 12 family members (Table 3 and Table S3). In *Wolbachia* endosymbiont of *Culex quinquefasciatus*, we detected the Cell division protein *FtsI/penicillin-binding protein 2* (COG0768). Finally, we found the COG Dehydrogenases with different specificities (related to short-chain alcohol dehydrogenases, COG1028) in *Anaplasma centrale str. Israel*. Positive substrates were searched in the other organisms of the family to identify orthologs proteins (when present) to analyze possible modifications of the signal peptides. Orthologs for the protein COG1028 identified in *Anaplasma centrale str. Israel* were found in all other genome selected. *A. centrale* has a typical Tat-like signal peptide conformed by the RR and consensus amino acids; interestingly, other organisms from the family showed several modifications with one or both R replaced by lysine (RK, KR or KK) (Figure S2). In addition *A. marginale str. St. Maries* and *A. marginale str. Florida* were annotated starting upstream in comparison to the others orthologs, which in turn would affect the Tat signal prediction. The *penicillin-binding protein* (COG0768) was identified as a positive substrate by two different software programs (TatP and TATFIND) in *Wolbachia* endosymbiont of *Culex quinquefasciatus*, but was positive only for TATFIND in the other three *Wolbachia sp.* studied. Orthologs proteins were identified only in *A. marginale* (*str. St. Maries* and *str. Florida*) and *A. centrale Israel*, which showed conserved blocks along the protein and the characteristic amino acids from the signal peptide (RR). However, the signal peptide has a substitution in the position next to RR (an Isoleucine instead of Serine or Alanine), which prevented the recognition as true substrate by TATFIND algorithm. (Figure S3).

**Table 3:** COG definition for Tat predicted substrates in *Anaplasmataceae* family^a^.

*Anaplasmataceae* family (12 genomes)			
COG	GOG definition	Present in *Anaplasmataceae*	Present in *Brucellaceae*
COG0723	Rieske Fe-S protein	8 of 12	yes
COG0768	Cell division protein FtsI/penicillin-binding	1 of 12	no
COG1028	Dehydrogenases with different specificities	1 of 12	no

a Proteins positive with at least two of the three software programs were consider as Tat substrates.

The search in *Brucellaceae* organisms (10 complete genomes) for potential Tat substrates yielded 250 proteins positive for at least two software programs that could be clustered in 22 COGs (Table 4 and Table S4) with different levels of representation within the family, and seven COGs that were unique to *Ochrobactrum anthropi*. Among candidates, 14 were hypothetical proteins with no related COGs (Table S4). It is interesting to note in Table 4 the large number of periplasmic components of solute-binding proteins likely to be dependent on Tat export. In this regard, the presence of Tat-like signal peptides in the periplasmic components of ABC transporters has been previously reported in *Rhizobum leguminosarum bv. viciae* [32] and Halobacteraceae [54], [55]. Import systems are found only in prokaryotic organisms and contain both ABC domains and inner membrane domains, along with extra-cytoplasmic binding proteins (BPs) designed to bind the specific allocrite of that ABC system. In Gram-negative bacteria, the BPs are located in the periplasm [56]. ABC systems import a diverse range of substrates into the bacterial cell including peptides, polyamines, metal ions, amino acids, iron, and sulphates [56], [57]. We also identified the COG Nitrous oxide reductase (*Nos*; COG4263) potentially exported by the Tat system. Previous studies have described the role of the Tat machinery in nitrous oxide reductase translocation in *Pseudomonas stutzeri* [58], where the Tat system has been shown to be necessary for establishing anaerobic nitrite denitrification. *Nos* is one of the four *Brucella* spp. reductases involved in the “denitrification island” that allow bacteria to grow under low-oxygen tension inside macrophages by respiration of nitrate [59]. Finally, COG0723 (Rieske Fe-S protein) was the only category shared by *Anaplasmataceae* and *Brucellaceae* Tat-dependent secretome (Table 3 and Table 4).

**Table 4:** COG definition for Tat predicted substrates in *Brucellaceae* family^a^.

*Brucellaceae* family (10 genomes)			
COG	GOG definition	Present in *Brucellaceae*	Present in Rickettsiales
COG0723	Rieske Fe-S protein	10 of 10	yes
COG0747	ABC-type dipeptide transport system, PC	10 of 10	no
COG1651	DSBA oxidoreductase; thiol:disulfide interchange	10 of 10	yes
COG2132	Putative multicopper oxidases	10 of 10	no
COG2041	Sulfite oxidase and related enzymes	10 of 10	no
COG1376	Uncharacterized protein conserved in bacteria	10 of 10	no
COG4263	Nitrous oxide reductase	9 of 10	no
COG3206	Uncharacterized protein involved in exopolysaccharide biosynthesis	9 of 10	no
COG3319	Thioesterase domains of type I polyketide synthases or non-ribosomal peptide synthetases	9 of 10	no
COG3683	ABC-type uncharacterized transport system, PC	9 of 10	no
COG4134	ABC-type uncharacterized transport system, PC	9 of 10	no
COG4213	ABC-type xylose transport system, PC	8 of 10	no
COG1477	Membrane-associated lipoprotein involved in thiamine biosynthesis	8 of 10	no
COG0715	ABC-type nitrate/sulfonate/bicarbonate transport, PC	8 of 10	no
COG1464	ABC-type metal ion transport system, PC/surface antigen	8 of 10	no
COG4663	TRAP-type mannitol/chloroaromatic compound transport system, PC	8 of 10	no
COG2989	Uncharacterized protein conserved in bacteria	8 of 10	no
COG1574	Predicted metal-dependent hydrolase with the TIM-barrel	7 of 10	no
COG4166	ABC-type oligopeptide transport system, PC	5 of 10	no
COG2340	Uncharacterized protein with SCP/PR1 domains	5 of 10	no
COG0612	Predicted Zn-dependent peptidases	4 of 10	yes
COG0741	Soluble lytic murein transglycosylase and related regulatory proteins	2 of 10	yes
COG0243	Anaerobic dehydrogenases, selenocysteine-containing	Ochrobactrum	no
COG1879	ABC-type sugar transport system, PC	Ochrobactrum	no
COG2837	Predicted iron-dependent peroxidase	Ochrobactrum	no
COG0683	ABC-type branched-chain amino acid transport, PC	Ochrobactrum	no
COG0687	Spermidine/putrescine-binding PC	Ochrobactrum	no
COG3019	Predicted metal-binding protein	Ochrobactrum	no
COG3246	Uncharacterized conserved protein	Ochrobactrum	no

Abbreviations: PC: periplasmic protein.

a Proteins positive with at least two of the three software programs were consider as Tat substrates.

Tat predicted substrates with no related COGs are listed in Table S4.

## Discussion

This work is the first description of the Tat system in two important pathogens: *Anaplasma marginale* and *Brucella abortus*. We identified the Tat components and studied the conservation of structural features and genome organization of the *tatA*, *tatB* and *tatC* genes in organisms from the α-proteobacteria class. We analyzed the transcription patterns and stoichiometry ratios of *tat* mRNA and functionality under different *tat* gene organizations (operon vs. disperse) to study the impact of genomic and regulatory conservation on functionality. The use of the Tat system was analyzed using available prediction algorithms for the identification of the Tat signal peptide, to study a potential role of the protein export system in conferring adaptive skills or in the pathogenesis of these phylogenetic groups.

In the past years, rapid progress has been made in unraveling the molecular mechanism and biochemical characterization of the Tat system as an alternative translocation system in bacteria. Despite this progress, little is known concerning the Tat system relevance in the α-proteobacteria [30], [32], [60]–[62]. This group shows a great genome size variation (1–10 Mb) associated with massive gene expansions and extreme losses [63], diversity in lifestyles, ecological niches (from obligate intracellular to free living organisms) and infection strategies [3], which could be partially explained with a thorough understanding of the protein translocation systems and exported substrates as key players.

Fifty-three annotated genome sequences from the α-proteobacteria class were analyzed in this study. We confirmed the presence of the *tatA*, *tatB* and *tatC* genes for the assembly of the translocation machinery in almost all members. Our identification of the *tatA* gene in *A. marginale str. St. Maries* and *Ehrlichia ruminantium str. Gardel*, which was significantly shorter than its orthologs in the α-proteobacteria class, revealed that, in agreement with similar observations [6], short ORFs are frequently omitted by automated annotation methods, like those used for processing the genomes of both organisms [49], [50]. In addition, in some obligate intracellular bacteria that have undergone genomic reduction [3], the identification of proteins of multicomponent systems might be hampered when selection does not favor the clustering of genes within one operon. In this regard, the *tat* gene organization revealed a great diversity within the class. In most members of the genus, *tat* genes are typically arranged in the canonical structure, encoded by three genes in operon (*tatABC*). Conversely, the members of the order Rickettsiales, exposed to an extraordinary trend towards genome reduction, displayed a dispersed Tat translocation machinery organization, with the three genes scattered throughout the genome (Figure 1B). A dispersed organization for the *tat* genes has been previously described for *Rickettsia prowazekii* [11]. Given the process of genome reduction observed in the Rickettsiales, it could be argued that this mechanism caused the splitting of the Tat operon. However, at present we cannot rule out other rearrangement generating processes like recombination. The *succinate dehydrogenase* gene arrangement and expression has been recently studied in *Anaplasma phagocytophilum*, another genome-reduced bacterium [64]. In that work, the authors described an overall conservation of *sdh* genes and critical amino acids, suggesting that these subunits remain functional. However, this bacterium showed an unusual genomic rearrangement, expression and operon splitting pattern. Interestingly, some split genes alternatively presented ATG or GTG start codons as well as the presence or absence of Shine-Dalgarno (SD) sequences, which may represent alternative mechanisms to control gene expression in fragmented operons. Several studies have described an atypical nature of the bacterial type IV secretion system (T4SS) in organisms from the Rickettsiales order [5], [6]. These studies have revealed a reduced T4SS as compared with *virB/virD* T4SS from *Agrobacterium tumefaciens*. Furthermore, the arrangement of Vir genes was non-canonically relative to the most frequently observed organizations, in which scattered genes are located in distant genome positions. In the rickettsial pathogen *Ehrlichia chaffeensis*, the *virBD* genes are split into two operons (*virB3–virB6* and *virB8*–*virD4*). Electrophoretic mobility shift assays revealed a previously unidentified protein that specifically binds to the promoter regions upstream the *virBD* loci and it has been proposed to regulate the five *virBD* loci to allow developmental stage-specific expression of the T4SS system in *E. chaffeensis* [5]. These results support the hypothesis of operon fragmentation events as a frequent phenomenon in obligate intracellular bacteria that suffered genomic rearrangements, where the loss of a coordinated expression to ensure equimolar amounts of each protein should require alternative mechanisms by which the organisms could coordinate the appropriate protein levels.

Recalling the phenomenon of gene loss events due to genome reduction, the absence of the *tatB* gene in *Rickettsia*, *Neorickettsia*, *Orientia* and *Wolbachia* could have led to an abrogation of the Tat system. However, it has been described that organisms such as gram-positive bacteria and archaea do not require TatB for a functional Tat translocase [15], [16] that is fully active as the TatAC-type complex [16]. In addition, a study in which some amino acids of TatA were replaced strongly suggests that the biological activity of TatA and TatB has been condensed into one protein in those systems that did not encode an obvious TatB protein [17]. The TatB protein is absent in *Rickettsia* spp., *Neorickettsia* spp., *Wolbachia* spp. and *Orientia* spp., in which the conservation of functionality has not been demonstrated yet, and thus further experimental work on this subject is required.

In spite of the scattered organization and smallest ORFs for *A. marginale*, sequence analysis indicated an overall conservation of essential amino acids, structural features and critical protein portions in both organisms, suggesting that functionality is conserved (Figure S1).

Experimental results in *A. marginale* demonstrated that the TatA subunit can fully restore Tat functionality in the heterologous system of *E coli*. In fact, in almost all cross-species complementation tests that have been assessed, TatA proteins always seem to retain some level of function in the heterologous host, suggesting that the constraints on TatA function are less severe than those on TatB or TatC [31], [40]. This is consistent with the role of the TatA subunit within the protein complex, where most interactions of the heterologously expressed TatA would be self-oligomerized to assemble into channel-forming multimers. By contrast, the constraints on cross-complementation with heterologously expressed TatB or TatC proteins are likely to be much more stringent since this process would require the recognition of non-native signal peptides of *E. coli*. Since TatB interacts with each of the other Tat components and with Tat signal peptides, cross-complementation with this subunit might be expected to be less efficient than that with other Tat proteins. TatB from *A. marginale* allowed significant growth of BOD-P on selective media containing TMAO, indicating Tat function. However, it failed to grow in SDS-containing media, probably due to a substrate-specific effect. The *tatC* gene of *A. marginale* completely failed to complement BILK0-P in different selective media. We were not able to demonstrate whether the *A. marginale* TatC protein was expressed in these experiments due to the lack of a native antibody against the protein; however, we corroborated the expression of the *tatA*, *tatB* and *tatC* genes from *A. marginale* in the complemented *E. coli* strains by RT-PCR (data not shown). Taken together, our results suggest that *A. marginale* conserved a functional Tat system, since TatA and TatB were able to restore functionality. In spite of its conservation of structure and essential amino acids, TatC was not able to restore functionality in the heterologous system. Considering that TatC has been implicated as a specificity determinant for Tat-dependent secretion through the recognition of Tat signal peptides [2], [65] and that the *A. marginale* genome does not encode for any of the Tat substrates involved in the experimental tests used in this study, negative results could be related to the inability to recognize Tat signal peptides from *E. coli* Tat native substrates. Another possible explanation could be an anomalous (if any) interaction due to the heterologous nature of the complex (Table 2). However, the existence of putative co-evolution between *A. marginale* (and related organisms) Tat signal peptides and the machinery for the specific recognition is an interesting question that has not been addressed yet.

The three subunits of *B. abortus* were able to restore Tat function, suggesting a complete conservation of functionality and substrate recognition (Figures 4C, 4D, 5 and 6), with the exception of the SDS selective medium (Figure 4A, 4B, and Table 2).

The mRNA transcript levels obtained for *tat* genes in *A. marginale* correlate with the described stoichiometry of the TatABC protein complex (20–30∶1∶1) [2], [65]. Furthermore, it could represent an indirect evidence not only of potential functionality of the system, but also of an alternative transcriptional regulatory mechanism to operon organization, that will require further experiments to test if mRNA abundance difference correlates with a equivalent protein difference. For *B. abortus*, we demonstrated that the components are transcribed in polycistronic mRNA. Moreover, equal amounts of mRNA were detected for each gene, in agreement with that expected for operon expression system regulation that relies on post-transcriptional mechanisms to end up with the appropriate relative amounts of proteins according to the correct stoichiometry of the multicomponent system.

Although *Brucellaceae* and *Anaplasmataceae* are phylogenetically related groups, there were significant differences in their predicted Tat secretome. Our data are consistent with previous analysis of Proteobacteria, in which, regardless of the phylogeny, pathogenic bacteria appear as poor users of Tat, while the free-living and soil bacteria are moderate-to-extensive users [66]. This characteristic, which links Tat usage to an organism's lifestyle, is clearly shown in the *Brucellaceae* family, where *Ochrobactrum antropi* exhibited a significantly higher number of predicted Tat substrates than *Brucella* spp. (Table 4). In this regard, as facultative intracellular bacteria, *Brucella* spp. seems to be an intermediate stage between pathogens and free-living organisms. This hypothesis is supported by the relatively large amount of ABC transport machinery predicted as Tat substrate in *Brucellaceae*, high-affinity substrate binding proteins of transporters used to scavenge nutrients from competitive and variable habitats, although most of the time *Brucella* spp. can acquire nutrition from a stable niche.

Notably, both *Anaplasmataceae* and *Brucellaceae* exhibited ubiquinol-cytochrome c reductase iron-sulfur subunit (Rieske iron-sulfur domain-containing protein) as the only one shared COG predicted as Tat substrate. The Rieske Fe/S protein is an essential subunit of mitochondrial and bacterial bc1 complexes, which are central redox carriers in respiratory electron transport and belong to a class of Tat substrates that are integral membrane proteins with an uncleaved Tat signal peptide that functions as an N-terminal transmembrane anchor and a large domain periplasmically located. Importantly, it has been recently demonstrated that the Tat pathway is indispensable for correct integration of the signal peptide and anchoring of the periplasmic iron–sulfur domain to the membrane in the Gram-negative facultative intracellular lung pathogen *Legionella pneumophila* [67]. Furthermore, one of the predicted Tat substrates in *Anaplasmataceae* is Penicillin-binding protein 2 (PBP2- COG0768), a well-characterized class of enzymes required for the assembly of peptidoglycan from intracellularly synthesized precursors. Particularly, PBP2 function in assembling peptide cross-links and in rod-shaped bacteria is implicated in the elongation phase of cell growth [68]. Interestingly, though we detected a signal Tat peptide only in PBP2 from *Wolbachia*, the *A. marginale* orthologs were highly conserved (Figure S3). Additionally, we verified the absence of PBP2 orthologs in *Ehrlichia* spp., *Neorickettsia* spp. and *A. phagocytophilum* by BlastP and tBlastn searches. Although pathogenic bacteria of *Anaplasmataceae* family are expected to bear a low number of Tat substrates, we cannot rule out that the number of potential substrates is underestimated due to inaccurate determination of the start codon during the automatic annotation process (Figure S2 and Figure S3).

These results and observations provide new insights into the characterization of the Tat system and novel proteins potentially secreted by this translocation complex, to unravel their role in proving adaptive skills and intracellular infection strategies.

## Supporting Information

Figure S1 **TatA, B and C protein sequence alignments.** Multiple alignments of amino acid sequences from TatA, TatB and TatC proteins. Amino acid sequences from *A. marginale str. St. Maries*, *B. abortus 2308* and *E. coli K12* were aligned using CLUSTAL W [33] (A) TatA protein, (B) TatB protein, (C) TatC protein.
(DOC)

Figure S2 **COG1028,** ***Anaplasmataceae*** **protein sequence alignment.** Multiple alignment of orthologs amino acid sequences (Dehydrogenases with different specificities) using CLUSTAL W [33] from *Anaplasma centrale str. Israel, Anaplasma marginale str. Florida, Anaplasma marginale str. St. Maries, Anaplasma phagocytophilum HZ, Ehrlichia canis str. Jake, Ehrlichia chaffeensis str. Arkansas, Ehrlichia ruminantium str. Gardel, Ehrlichia ruminantium str. Welgevonden, Neorickettsia risticii str. Illinois, Neorickettsia sennetsu str. Miyayama, Wolbachia endosymbiont of Culex quinquefasciatus, Wolbachia endosymbiont of Drosophila mel, Wolbachia endosymbiont str. TRS Brugia malayi, Wolbachia sp. wRi*. Annotated start sites were highlighted in red, RR motif and its variants were highlighted in yellow.
(TIF)

Figure S3 **COG0768,** ***Anaplasmataceae*** **protein sequence alignment.** Multiple alignment of orthologs amino acid sequences (Cell division protein FtsI/penicillin-binding protein 2) using CLUSTAL W [33] from *Anaplasma centrale str. Israel, Anaplasma marginale str. Florida, Anaplasma marginale str. St. Maries, Wolbachia endosymbiont of Culex quinquefasciatus, Wolbachia endosymbiont of Drosophila mel, Wolbachia endosymbiont str. TRS Brugia malayi, Wolbachia sp. wRi*. Annotated start sites were highlighted in red, RR motif and its variants were highlighted in yellow.
(TIF)

Table S1 **Primers used for RT-PCR and Real-time qPCR.**
(DOC)

Table S2 **Selected α-proteobacteria genomes analyzed in the study (Accession numbers of 53 organisms).**
(DOC)

Table S3 **Tat predicted substrates for** ***Anaplasmataceae*** **family.**
(XLS)

Table S4 **Tat predicted substrates for** ***Brucellaceae*** **family.**
(XLS)
