# Supplementary material for: The Twin-Arginine Translocation Pathway in α-Proteobacteria Is Functionally Preserved Irrespective of Genomic and Regulatory Divergence
Source: PLoS One. 2012 Mar 15;7(3):e33605. doi: 10.1371/journal.pone.0033605 (PMC3305326; doi:10.1371/journal.pone.0033605)
Supplement: Figure S1 — TatA, B and C protein sequence alignments. Multiple alignments of amino acid sequences from TatA, TatB and TatC proteins. Amino acid sequences from A. marginale str. St. Maries, B. abortus 2308 and E. coli K12 were aligned using CLUSTAL W [33] (A) TatA protein, (B) TatB protein, (C) TatC protein. (DOC) [file pone.0033605.s001.doc]

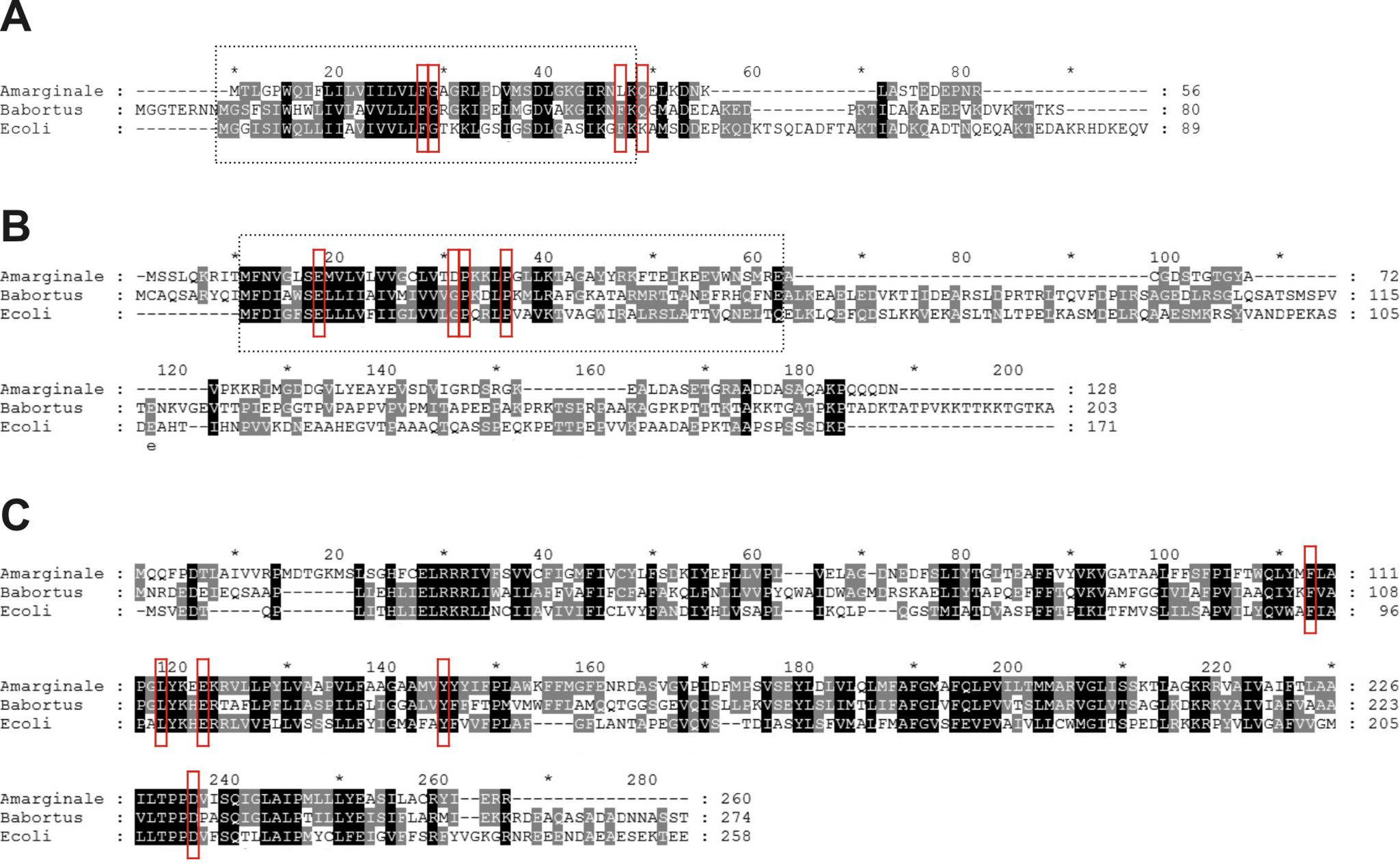


**Figure S1. TatA, B and C protein sequence alignment.**

Multiple alignment of amino acid sequences from TatA, TatB and TatC proteins. Amino acid sequences from *A. marginale St. Maries*, *B. abortus 2308* and *E. coli K12* were aligned using CLUSTAL W [1] **(A)** TatA protein, **(B)** TatB protein, **(C)** TatC protein.

The main differences between TatA and TatB proteins are associated with protein length, the predicted hinge region and conservation of amphipathic helical regions. The *tatA* and *tatB* genes are truncated in Rickettsiales though maintaining essential functional domains in the first 40 aminoacids. For TatA, the F20 and G21 amino acids, described to participate in the hinge region, are strictly conserved. However, F39, postulated as essential for Tat activity is replaced by another non-polar leucine in *A. marginale*. K41 in *E. coli* is replaced in both organisms by glutamine, which is a polar but uncharged residue. The overall organization of TatB seems to retain essential domains although we can see different important substitutions. The transmembrane domain remains highly conserved; including the invariant glutamic acid residue in the transmembrane domain (E8), proposed to have a role in proton translocation [2]. Both conserved prolines, proposed to reinforce the hinge region, are conserved [3]. Glycine21, described as necessary to support an efficient translocation [3], is replaced by isoleucine in *A. marginale*. The amphipathic is less conserved than the N-terminal domains but the pattern of the three glutamic acids seems to be conserved. Multiple sequence analyses indicate that TatC is highly conserved in all the genomes studied, conserving multiple putative transmembrane domains. F94, E103 and D211 determinants for Tat functionality are conserved [4] and L99A and Y126, which are important but non-essential for TatC activity, are also conserved [4].

**Additional References (Figure S1).**

1. Larkin MA, Blackshields G, Brown NP, Chenna R, McGettigan PA, et al. (2007) Clustal W and Clustal X version 2.0. Bioinformatics 23: 2947-2948.

2. Sargent F, Stanley NR, Berks BC, Palmer T (1999) Sec-independent protein translocation in Escherichia coli. A distinct and pivotal role for the TatB protein. J Biol Chem 274: 36073-36082.

3. Hicks MG, de Leeuw E, Porcelli I, Buchanan G, Berks BC, et al. (2003) The Escherichia coli twin-arginine translocase: conserved residues of TatA and TatB family components involved in protein transport. FEBS Lett 539: 61-67.

4. Buchanan G, de Leeuw E, Stanley NR, Wexler M, Berks BC, et al. (2002) Functional complexity of the twin-arginine translocase TatC component revealed by site-directed mutagenesis. Mol Microbiol 43: 1457-1470.
