# Supplementary figures and images for: The Twin-Arginine Translocation Pathway in α-Proteobacteria Is Functionally Preserved Irrespective of Genomic and Regulatory Divergence
Source: PLoS One. 2012 Mar 15;7(3):e33605. doi: 10.1371/journal.pone.0033605 (PMC3305326; doi:10.1371/journal.pone.0033605)

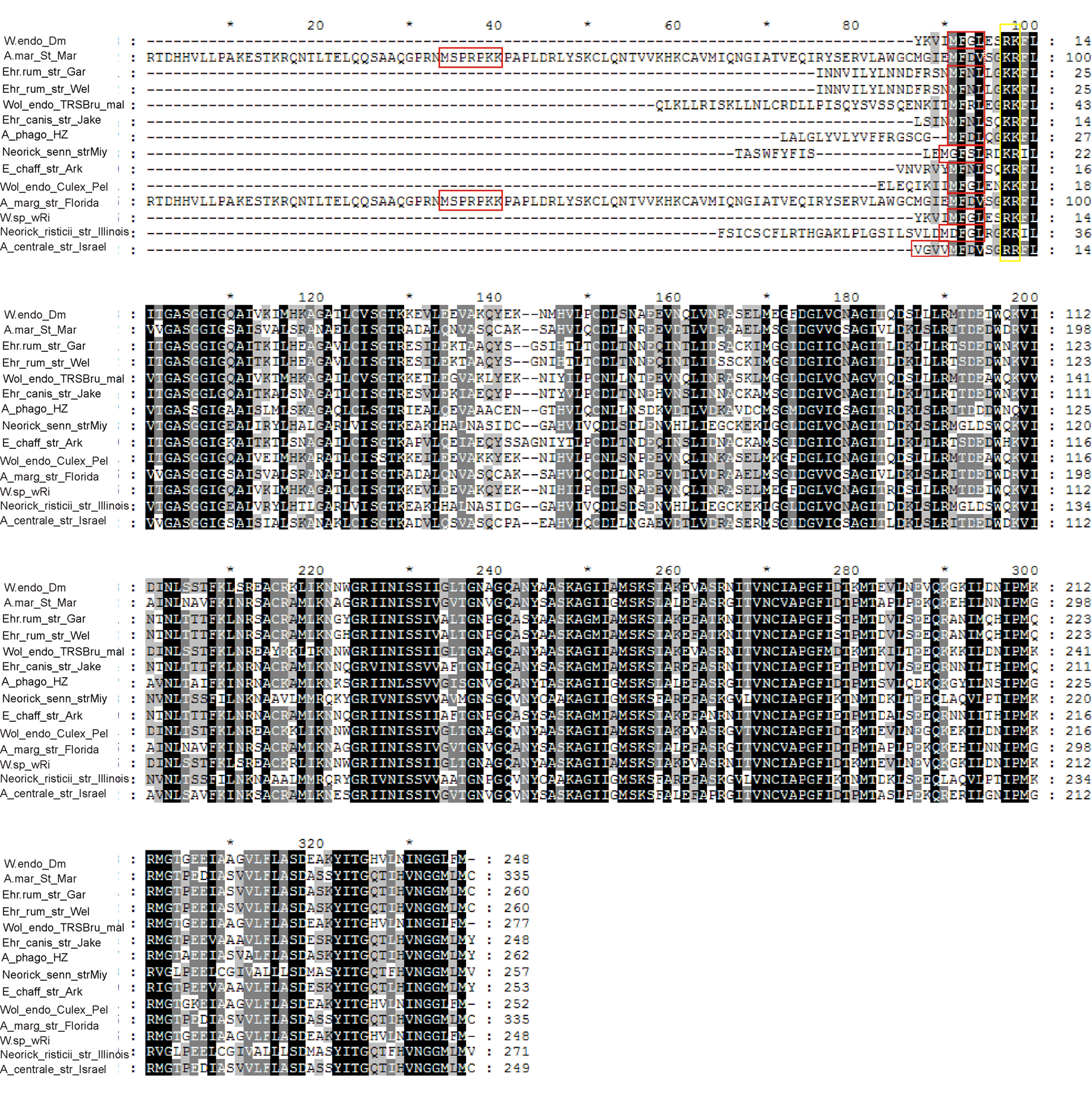

Supplement: Figure S2 — COG1028, Anaplasmataceae protein sequence alignment. Multiple alignment of orthologs amino acid sequences (Dehydrogenases with different specificities) using CLUSTAL W [33] from Anaplasma centrale str. Israel, Anaplasma marginale str. Florida, Anaplasma marginale str. St. Maries, Anaplasma phagocytophilum HZ, Ehrlichia canis str. Jake, Ehrlichia chaffeensis str. Arkansas, Ehrlichia ruminantium str. Gardel, Ehrlichia ruminantium str. Welgevonden, Neorickettsia risticii str. Illinois, Neorickettsia sennetsu str. Miyayama, Wolbachia endosymbiont of Culex quinquefasciatus, Wolbachia endosymbiont of Drosophila mel, Wolbachia endosymbiont str. TRS Brugia malayi, Wolbachia sp. wRi. Annotated start sites were highlighted in red, RR motif and its variants were highlighted in yellow. (TIF) [file pone.0033605.s002.tif]

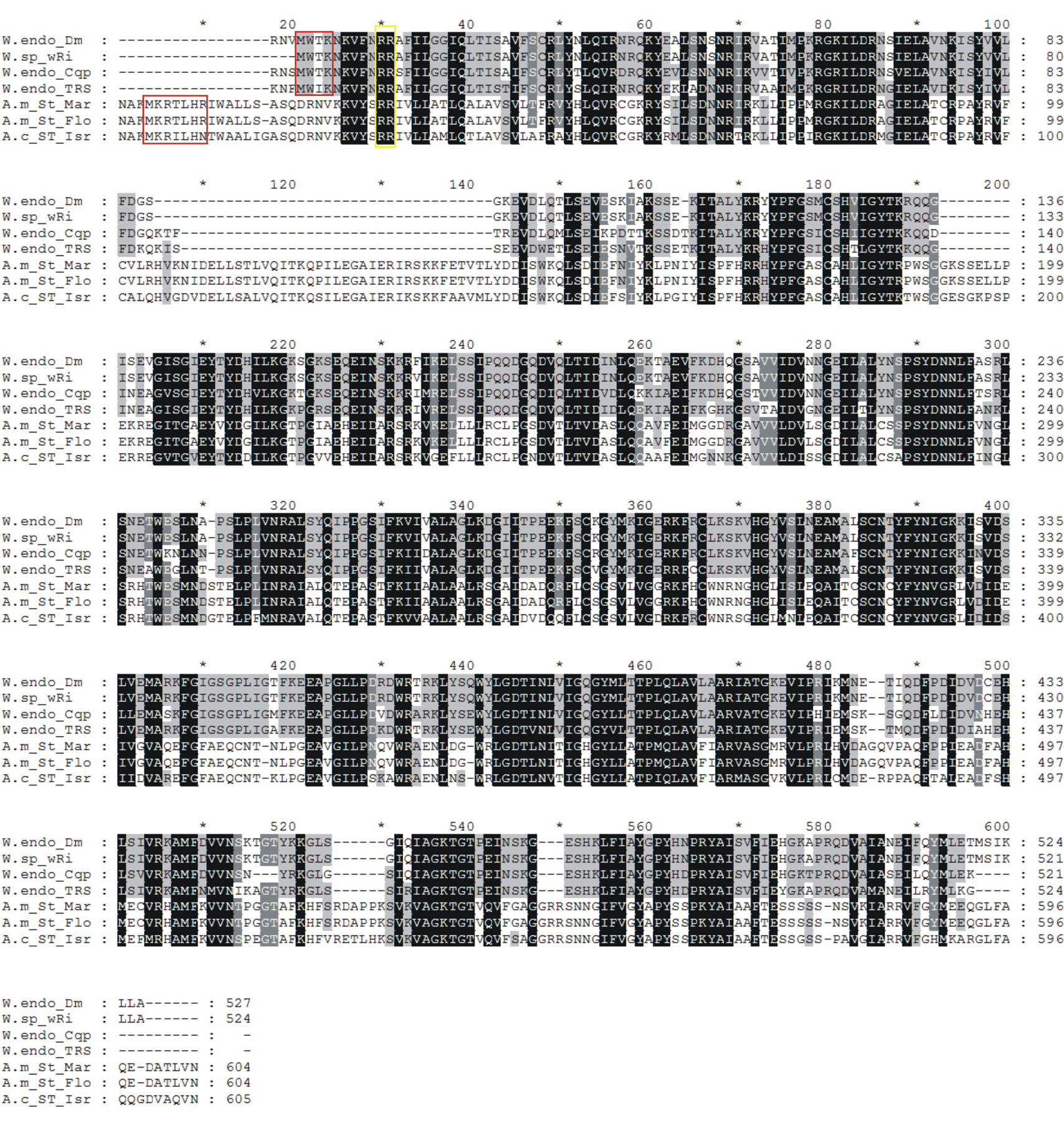

Supplement: Figure S3 — COG0768, Anaplasmataceae protein sequence alignment. Multiple alignment of orthologs amino acid sequences (Cell division protein FtsI/penicillin-binding protein 2) using CLUSTAL W [33] from Anaplasma centrale str. Israel, Anaplasma marginale str. Florida, Anaplasma marginale str. St. Maries, Wolbachia endosymbiont of Culex quinquefasciatus, Wolbachia endosymbiont of Drosophila mel, Wolbachia endosymbiont str. TRS Brugia malayi, Wolbachia sp. wRi. Annotated start sites were highlighted in red, RR motif and its variants were highlighted in yellow. (TIF) [file pone.0033605.s003.tif]
