# Supplementary material for: The Twin-Arginine Translocation Pathway in α-Proteobacteria Is Functionally Preserved Irrespective of Genomic and Regulatory Divergence
Source: PLoS One. 2012 Mar 15;7(3):e33605. doi: 10.1371/journal.pone.0033605 (PMC3305326; doi:10.1371/journal.pone.0033605)
Supplement: Table S1 — Primers used for RT-PCR and Real-time qPCR. (DOC) [file pone.0033605.s004.doc]

| **Table S1. Primers used for RT-PCR and Real-time qPCR.** | |
| --- | --- |
| **Primer designation** | **Sequence (5’ - 3’)** |
| RTTatAAMF | GGTCCGTGGCAGATTTTTCTT |
| RTTatAAMR | CTCAGACTACGGATGCCCTTTC |
| RTTatBAMF | ACGAGGCTTACGAAGTTAGCGA |
| RTTatBAMR | TTGGCTTGTGCAGAGGCAT |
| RTTatCAMF | CTCCGTCGTGTGTTTTATCGG |
| RTTatCAMR | TTATCGCCTGCAAGCTCCA |
| RT16SF | TGATCCTGGCTCAGAACGAAC |
| RT16SR | ACCCGTCTGCCACTAACCATAC |
| RTGroELF | GGCAAGATAGCACAGTGCGTAA |
| RTGroELR | TACCGTCAGTCCTTTCGACCTC |
| RTTatABruF | TGATCGTTCTGGCGGTTGT |
| RTTatABruR | CCATGCCCTGCTTGAAATTC |
| RTTatBBruF | GTCATGATTGTGGTGGTCGGT |
| RTTatBBruR | TGATGGCGAAACTCGTTGG |
| RTTatCBruF | TTTGCTTTTGGCCTGGTCTT |
| RTTatCBruR | GCATATTTGCGCTTGTCCTTC |
| IF1F | ACTGCTGCCCAATGCAATG |
| IF2R | CCAGAACACGGATACGGTTCTT |
| RPLLF | TGAGCTGTCCAAGCTTCTCGA |
| RPLLR | GAATTCGGTCTTTTCTTCTGCG |
