# Supplementary material for: The Twin-Arginine Translocation Pathway in α-Proteobacteria Is Functionally Preserved Irrespective of Genomic and Regulatory Divergence
Source: PLoS One. 2012 Mar 15;7(3):e33605. doi: 10.1371/journal.pone.0033605 (PMC3305326; doi:10.1371/journal.pone.0033605)
Supplement: Table S2 — Selected α-proteobacteria genomes analyzed in the study (Accession numbers of 53 organisms). (DOC) [file pone.0033605.s005.doc]

| **Table S2. 53 α-proteobacteria organisms analyzed in the study.** | |
| --- | --- |
| **RefSeq Genome ID** | **Organism** |
| NC_010161 | *Bartonella tribocorum CIP 105476* |
| NC_005956 | *Bartonella henselae str. Houston-1* |
| NC_008783 | *Bartonella bacilliformis KC583* |
| NC_006932 | *Brucella abortus bv. 1 str. 9-941* |
| NC_003317 | *Brucella melitensis 16M* |
| NC_004310 | *Brucella suis 1330* |
| NC_002678 | *Mesorhizobium loti MAFF303099* |
| NC_003047 | *Sinorhizobium meliloti 1021* |
| NC_003062 | *Agrobacterium tumefaciens str. C58* |
| NC_007761 | *Rhizobium etli CFN 42* |
| NC_008380 | *Rhizobium leguminosarum bv. viciae 3841* |
| NC_010725 | *Methylobacterium populi BJ001* |
| NC_010505 | *Methylobacterium radiotolerans JCM 2831* |
| NC_010581 | *Beijerinckia indica subsp. indica ATCC 9039* |
| NC_009720 | *Xanthobacter autotrophicus Py2* |
| NC_009937 | *Azorhizobium caulinodans ORS 571* |
| NC_004463 | *Bradyrhizobium japonicum USDA 110* |
| NC_007778 | *Rhodopseudomonas palustris HaA2* |
| NC_007406 | *Nitrobacter winogradskyi Nb-255* |
| NC_009719 | *Parvibaculum lavamentivorans DS-1* |
| NC_002696 | *Caulobacter crescentus CB15* |
| NC_008347 | *Maricaulis maris MCS10* |
| NC_008358 | *Hyphomonas neptunium ATCC 15444* |
| NC_003911 | *Silicibacter pomeroyi DSS-3* |
| NC_008209 | *Roseobacter denitrificans OCh 114* |
| NC_007493 | *Rhodobacter sphaeroides 2.4.1* |
| NC_008686 | *Paracoccus denitrificans PD1222* |
| NC_006526 | *Zymomonas mobilis subsp. mobilis ZM4* |
| NC_008048 | *Sphingopyxis alaskensis RB2256* |
| NC_007722 | *Erythrobacter litoralis HTCC2594* |
| NC_007794 | *Novosphingobium aromaticivorans DSM 12444* |
| NC_006677 | *Gluconobacter oxydans 621H* |
| NC_009484 | *Acidiphilium cryptum JF-5* |
| NC_007643 | *Rhodospirillum rubrum ATCC 11170* |
| NC_007626 | *Magnetospirillum magneticum AMB-1* |
| NC_007205 | *Candidatus Pelagibacter ubique HTCC1062* |
| NC_010263 | *Rickettsia rickettsii str. Iowa* |
| NC_003103 | *Rickettsia conorii str. Malish 7* |
| NC_009900 | *Rickettsia massiliae MTU5* |
| NC_006142 | *Rickettsia typhi str. Wilmington* |
| NC_000963 | *Rickettsia prowazekii str. Madrid E* |
| NC_009879 | *Rickettsia canadensis str. McKiel* |
| NC_009883 | *Rickettsia bellii OSU 85-389* |
| NC_009488 | *Orientia tsutsugamushi str. Boryong* |
| NC_007798 | *Neorickettsia sennetsu str. Miyayama* |
| NC_006833 | *Wolbachia endosymbiont strain TRS of Brugia malayi* |
| NC_002978 | *Wolbachia endosymbiont of Drosophila melanogaster* |
| NC_004842 | *Anaplasma marginale str. St. Maries* |
| NC_007797 | *Anaplasma phagocytophilum HZ* |
| NC_006831 | *Ehrlichia ruminantium str. Gardel* |
| NC_007354 | *Ehrlichia canis str. Jake* |
| NC_007799 | *Ehrlichia chaffeensis str. Arkansas* |
| NC_010473 | *Escherichia coli str. K12 substr. DH10B* |
